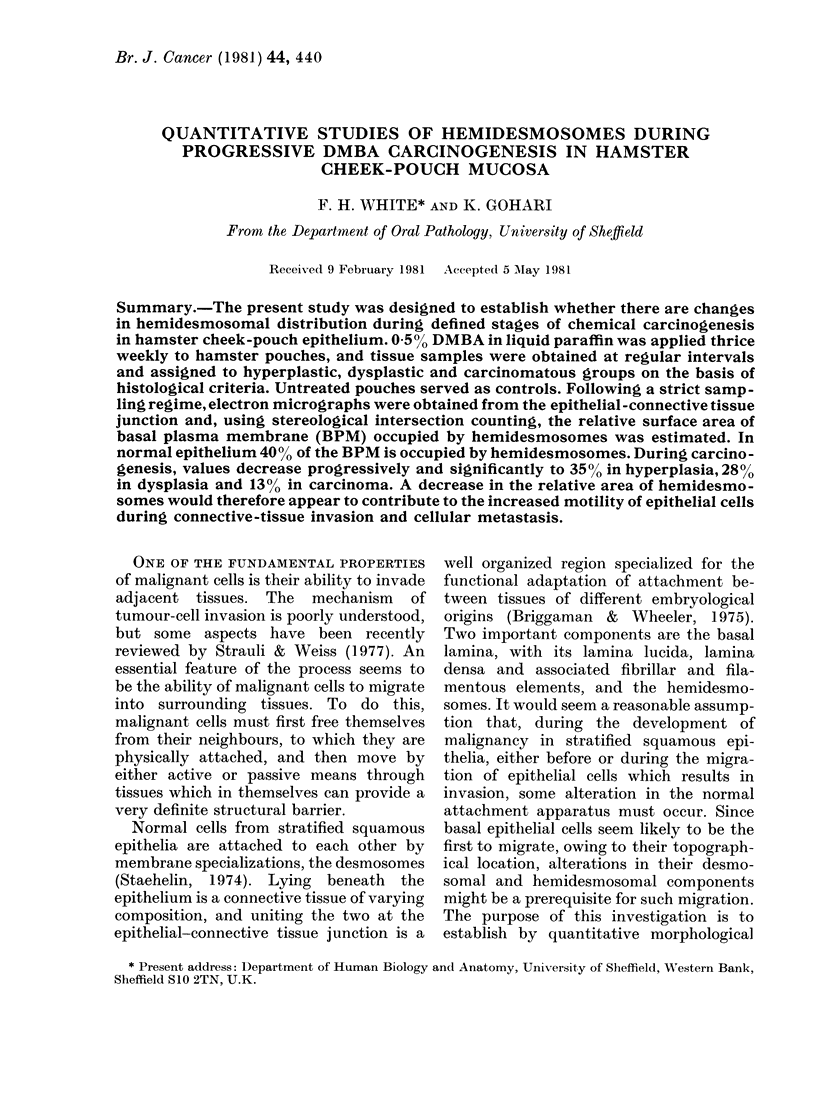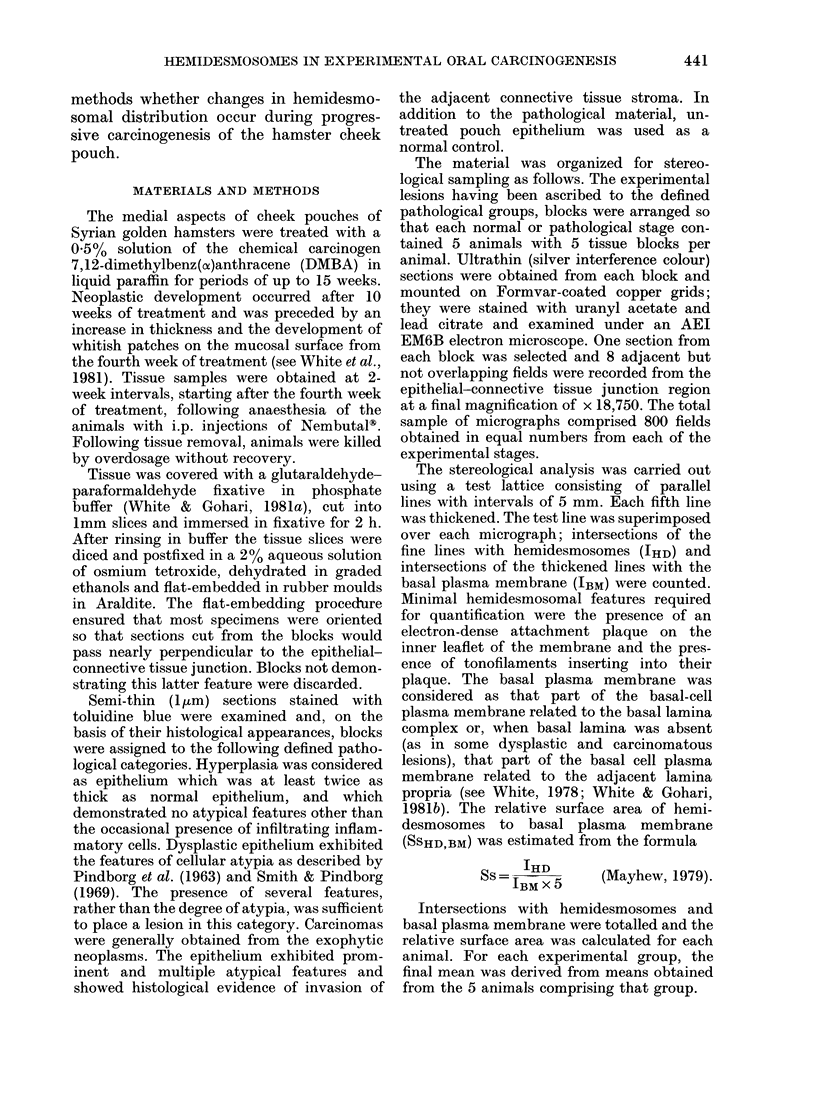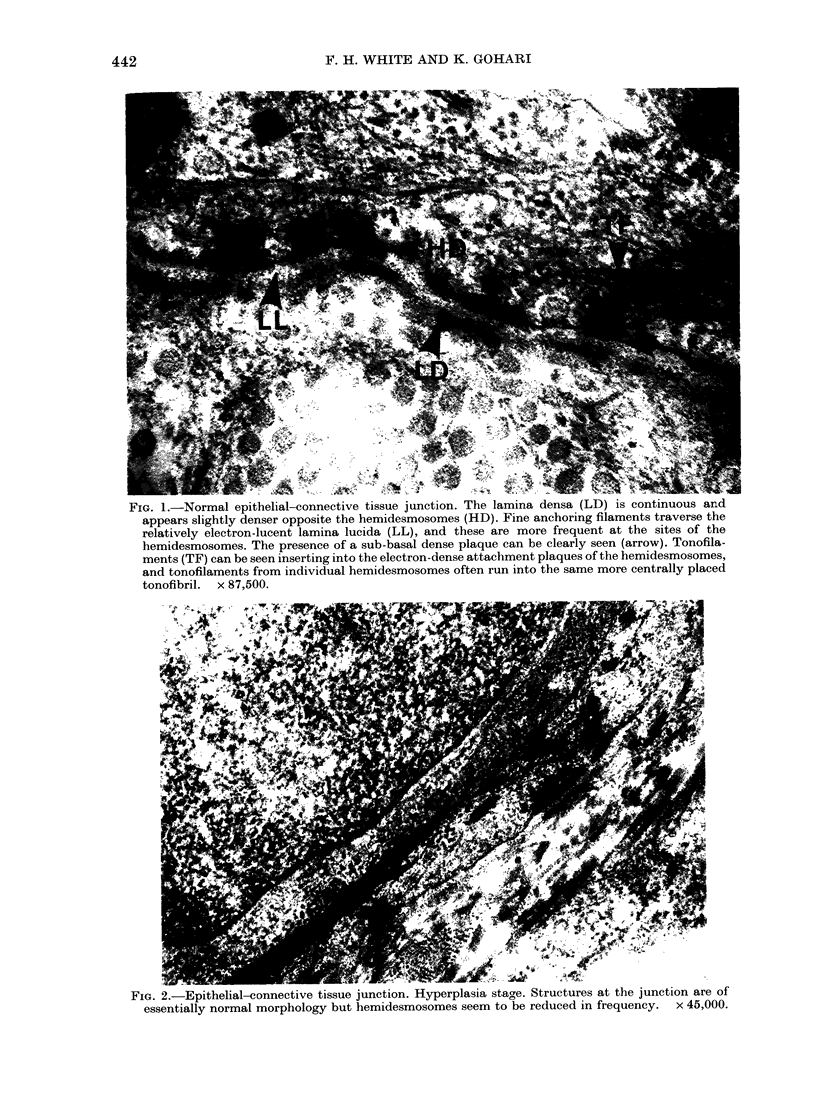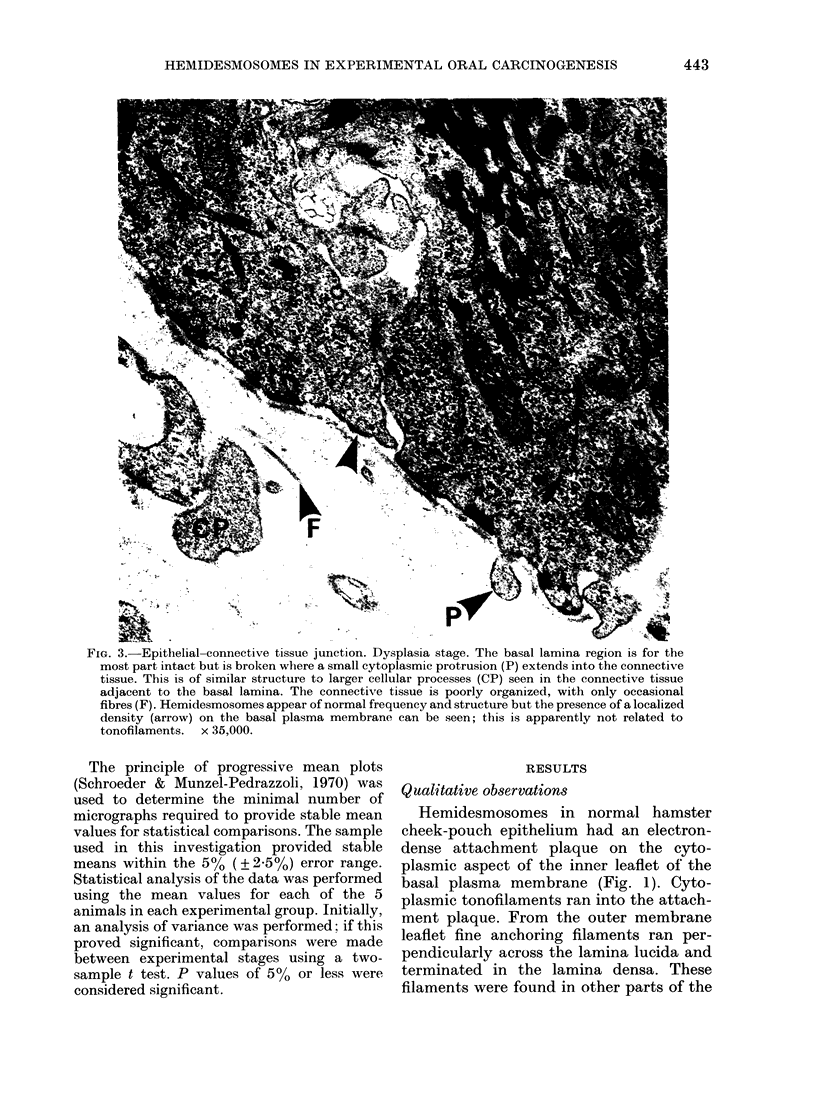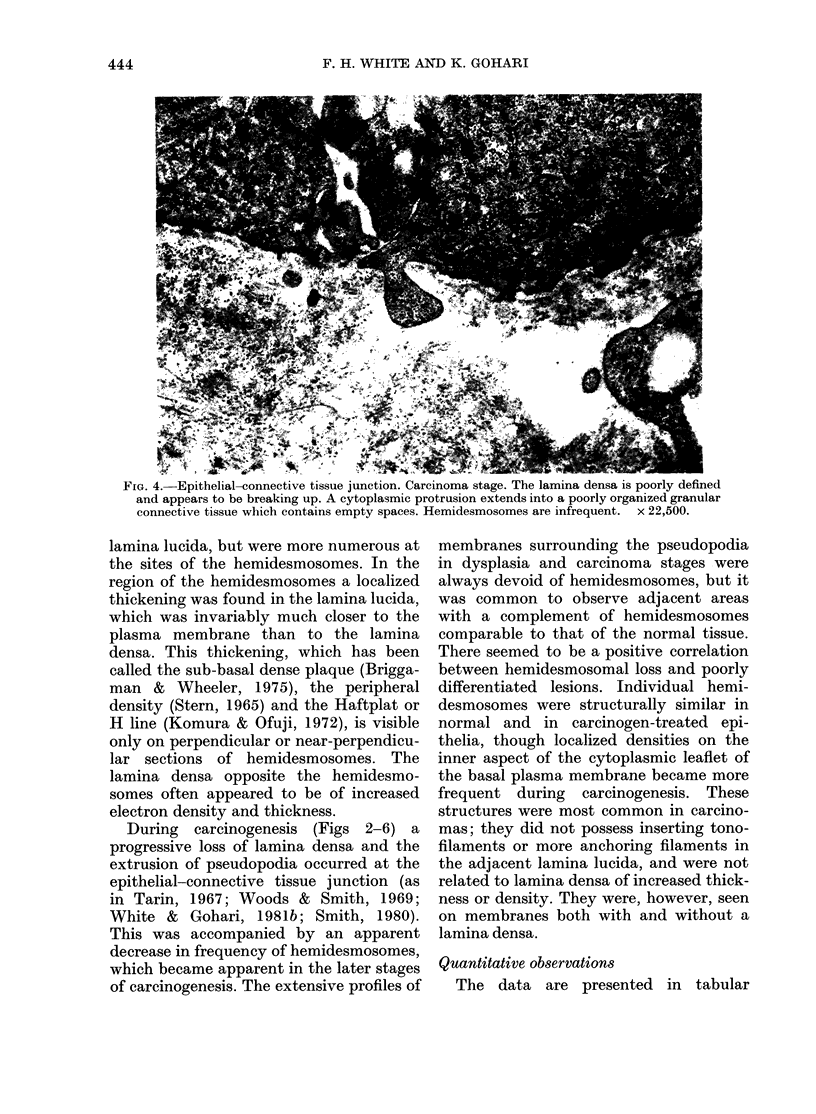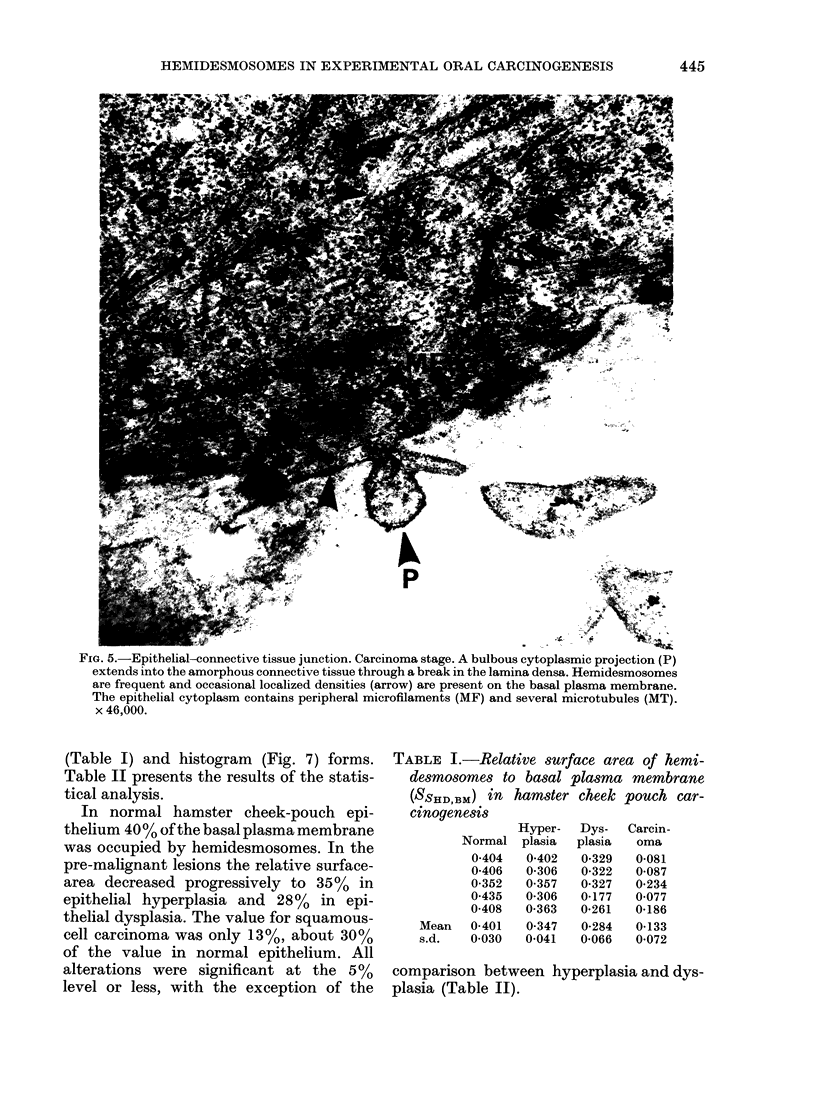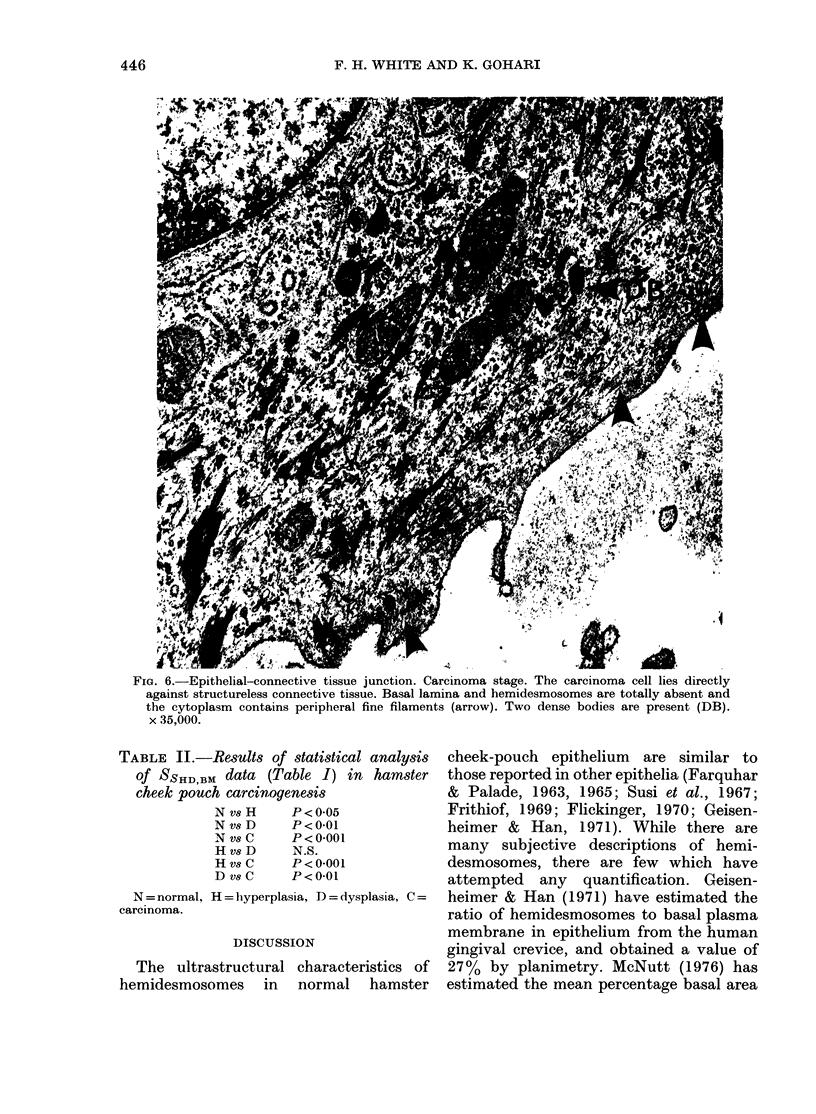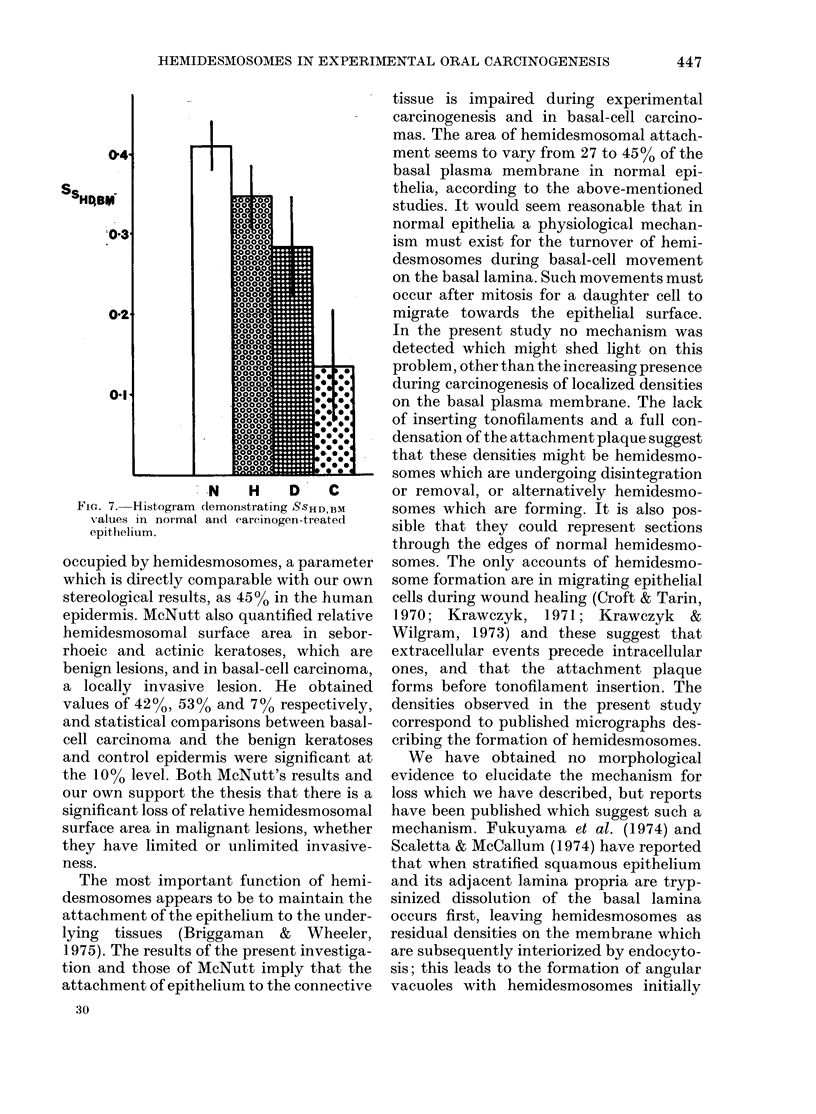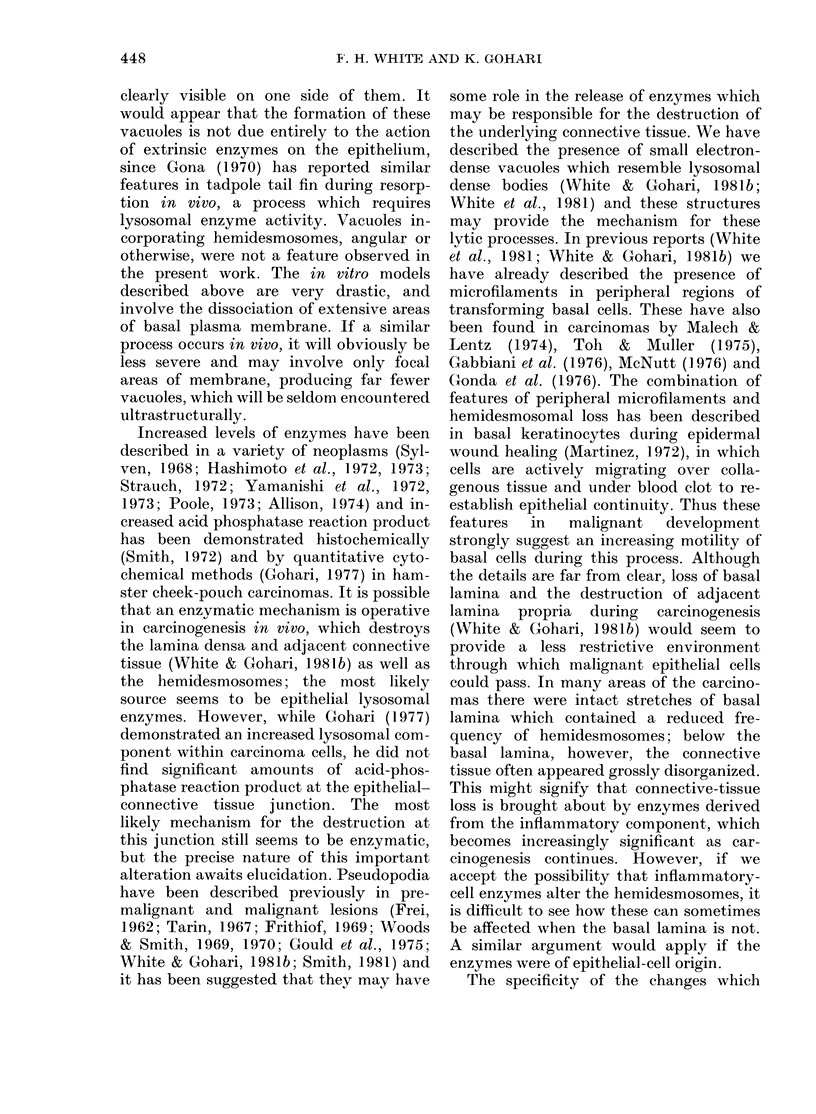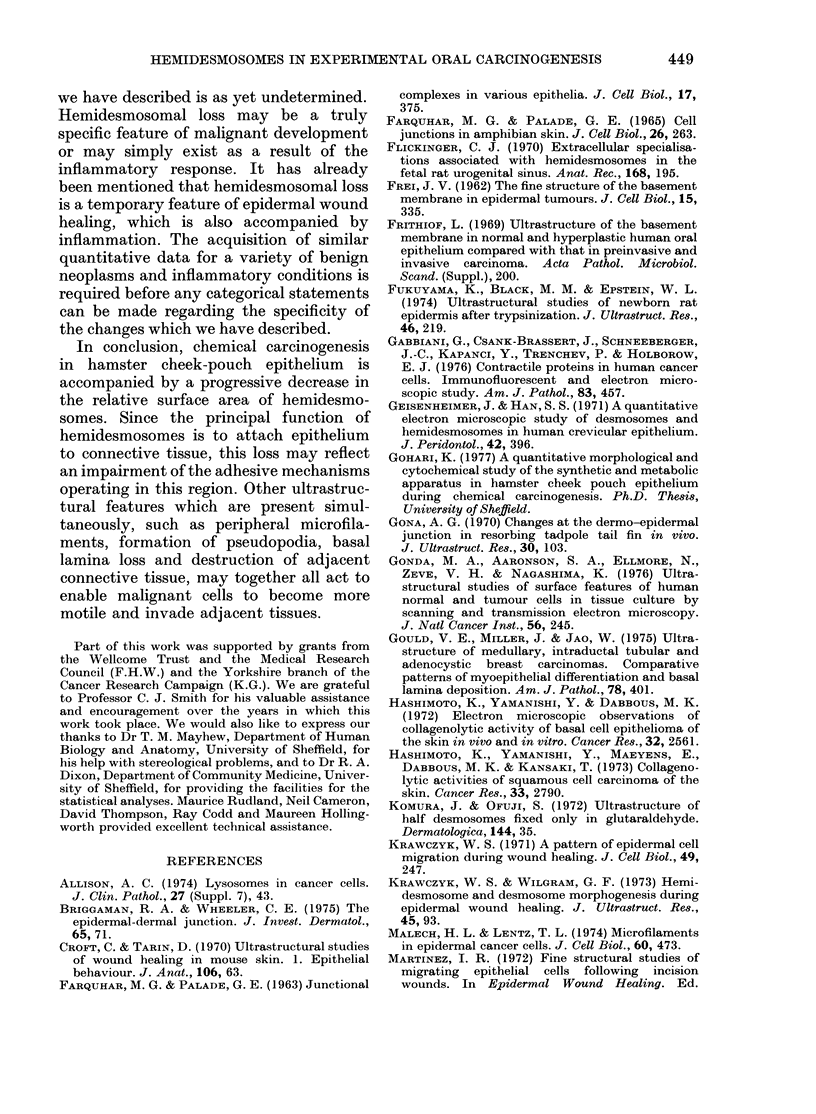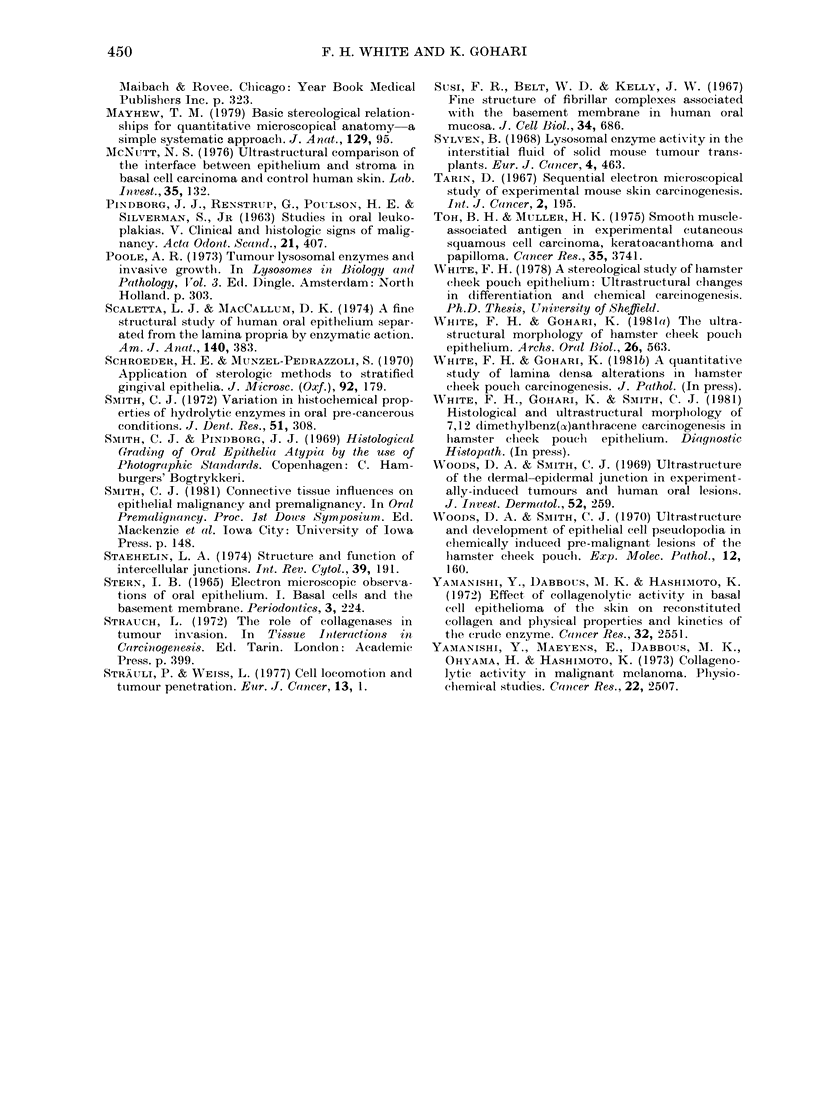# Quantitative studies of hemidesmosomes during progressive DMBA carcinogenesis in hamster cheek-pouch mucosa.

**DOI:** 10.1038/bjc.1981.203

**Published:** 1981-09

**Authors:** F. H. White, K. Gohari

## Abstract

**Images:**


*Br. J. Cancer* (1981) **44**, 440

### QUANTITATIVE STUDIES OF HEMIDESMOSOMES DURING PROGRESSIVE DMBA CARCINOGENESIS IN HAMSTER CHEEK-POUCH MUCOSA

F. H. WHITE* AND K. GOHARI

*From the Department of Oral Pathology, University of Sheffield*

Received 9 February 1981 Accepted 5 May 1981

**Summary.—The present study was designed to establish whether there are changes in hemidesmosomal distribution during defined stages of chemical carcinogenesis in hamster cheek-pouch epithelium. 0·5% DMBA in liquid paraffin was applied thrice weekly to hamster pouches, and tissue samples were obtained at regular intervals and assigned to hyperplastic, dysplastic and carcinomatous groups on the basis of histological criteria. Untreated pouches served as controls. Following a strict sampling regime, electron micrographs were obtained from the epithelial-connective tissue junction and, using stereological intersection counting, the relative surface area of basal plasma membrane (BPM) occupied by hemidesmosomes was estimated. In normal epithelium 40% of the BPM is occupied by hemidesmosomes. During carcinogenesis, values decrease progressively and significantly to 35% in hyperplasia, 28% in dysplasia and 13% in carcinoma. A decrease in the relative area of hemidesmosomes would therefore appear to contribute to the increased motility of epithelial cells during connective-tissue invasion and cellular metastasis.**

ONE OF THE FUNDAMENTAL PROPERTIES of malignant cells is their ability to invade adjacent tissues. The mechanism of tumour-cell invasion is poorly understood, but some aspects have been recently reviewed by Strauli & Weiss (1977). An essential feature of the process seems to be the ability of malignant cells to migrate into surrounding tissues. To do this, malignant cells must first free themselves from their neighbours, to which they are physically attached, and then move by either active or passive means through tissues which in themselves can provide a very definite structural barrier.

Normal cells from stratified squamous epithelia are attached to each other by membrane specializations, the desmosomes (Staehelin, 1974). Lying beneath the epithelium is a connective tissue of varying composition, and uniting the two at the epithelial–connective tissue junction is a well organized region specialized for the functional adaptation of attachment between tissues of different embryological origins (Briggaman & Wheeler, 1975). Two important components are the basal lamina, with its lamina lucida, lamina densa and associated fibrillar and filamentous elements, and the hemidesmosomes. It would seem a reasonable assumption that, during the development of malignancy in stratified squamous epithelia, either before or during the migration of epithelial cells which results in invasion, some alteration in the normal attachment apparatus must occur. Since basal epithelial cells seem likely to be the first to migrate, owing to their topographical location, alterations in their desmosomal and hemidesmosomal components might be a prerequisite for such migration. The purpose of this investigation is to establish by quantitative morphological

* Present address: Department of Human Biology and Anatomy, University of Sheffield, Western Bank, Sheffield S10 2TN, U.K.

methods whether changes in hemidesmosomal distribution occur during progressive carcinogenesis of the hamster cheek pouch.

### MATERIALS AND METHODS

The medial aspects of cheek pouches of Syrian golden hamsters were treated with a 0·5% solution of the chemical carcinogen 7,12-dimethylbenz(α)anthracene (DMBA) in liquid paraffin for periods of up to 15 weeks. Neoplastic development occurred after 10 weeks of treatment and was preceded by an increase in thickness and the development of whitish patches on the mucosal surface from the fourth week of treatment (see White *et al.*, 1981). Tissue samples were obtained at 2-week intervals, starting after the fourth week of treatment, following anaesthesia of the animals with i.p. injections of Nembutal®. Following tissue removal, animals were killed by overdosage without recovery.

Tissue was covered with a glutaraldehyde–paraformaldehyde fixative in phosphate buffer (White & Gohari, 1981*a*), cut into 1mm slices and immersed in fixative for 2 h. After rinsing in buffer the tissue slices were diced and postfixed in a 2% aqueous solution of osmium tetroxide, dehydrated in graded ethanols and flat-embedded in rubber moulds in Araldite. The flat-embedding procedure ensured that most specimens were oriented so that sections cut from the blocks would pass nearly perpendicular to the epithelial–connective tissue junction. Blocks not demonstrating this latter feature were discarded.

Semi-thin (1$\mu$m) sections stained with toluidine blue were examined and, on the basis of their histological appearances, blocks were assigned to the following defined pathological categories. Hyperplasia was considered as epithelium which was at least twice as thick as normal epithelium, and which demonstrated no atypical features other than the occasional presence of infiltrating inflammatory cells. Dysplastic epithelium exhibited the features of cellular atypia as described by Pindborg *et al.* (1963) and Smith & Pindborg (1969). The presence of several features, rather than the degree of atypia, was sufficient to place a lesion in this category. Carcinomas were generally obtained from the exophytic neoplasms. The epithelium exhibited prominent and multiple atypical features and showed histological evidence of invasion of the adjacent connective tissue stroma. In addition to the pathological material, untreated pouch epithelium was used as a normal control.

The material was organized for stereological sampling as follows. The experimental lesions having been ascribed to the defined pathological groups, blocks were arranged so that each normal or pathological stage contained 5 animals with 5 tissue blocks per animal. Ultrathin (silver interference colour) sections were obtained from each block and mounted on Formvar-coated copper grids; they were stained with uranyl acetate and lead citrate and examined under an AEI EM6B electron microscope. One section from each block was selected and 8 adjacent but not overlapping fields were recorded from the epithelial–connective tissue junction region at a final magnification of × 18,750. The total sample of micrographs comprised 800 fields obtained in equal numbers from each of the experimental stages.

The stereological analysis was carried out using a test lattice consisting of parallel lines with intervals of 5 mm. Each fifth line was thickened. The test line was superimposed over each micrograph; intersections of the fine lines with hemidesmosomes ($I_{HD}$) and intersections of the thickened lines with the basal plasma membrane ($I_{BM}$) were counted. Minimal hemidesmosomal features required for quantification were the presence of an electron-dense attachment plaque on the inner leaflet of the membrane and the presence of tonofilaments inserting into their plaque. The basal plasma membrane was considered as that part of the basal-cell plasma membrane related to the basal lamina complex or, when basal lamina was absent (as in some dysplastic and carcinomatous lesions), that part of the basal cell plasma membrane related to the adjacent lamina propria (see White, 1978; White & Gohari, 1981*b*). The relative surface area of hemidesmosomes to basal plasma membrane ($Ss_{HD,BM}$) was estimated from the formula

$$Ss = \frac{I_{HD}}{I_{BM} \times 5} \qquad \text{(Mayhew, 1979).}$$

Intersections with hemidesmosomes and basal plasma membrane were totalled and the relative surface area was calculated for each animal. For each experimental group, the final mean was derived from means obtained from the 5 animals comprising that group.

FIG. 1.—Normal epithelial–connective tissue junction. The lamina densa (LD) is continuous and appears slightly denser opposite the hemidesmosomes (HD). Fine anchoring filaments traverse the relatively electron-lucent lamina lucida (LL), and these are more frequent at the sites of the hemidesmosomes. The presence of a sub-basal dense plaque can be clearly seen (arrow). Tonofilaments (TF) can be seen inserting into the electron-dense attachment plaques of the hemidesmosomes, and tonofilaments from individual hemidesmosomes often run into the same more centrally placed tonofibril. ×87,500.

FIG. 2.—Epithelial–connective tissue junction. Hyperplasia stage. Structures at the junction are of essentially normal morphology but hemidesmosomes seem to be reduced in frequency. ×45,000.

FIG. 3.—Epithelial–connective tissue junction. Dysplasia stage. The basal lamina region is for the most part intact but is broken where a small cytoplasmic protrusion (P) extends into the connective tissue. This is of similar structure to larger cellular processes (CP) seen in the connective tissue adjacent to the basal lamina. The connective tissue is poorly organized, with only occasional fibres (F). Hemidesmosomes appear of normal frequency and structure but the presence of a localized density (arrow) on the basal plasma membrane can be seen; this is apparently not related to tonofilaments. × 35,000.

The principle of progressive mean plots (Schroeder & Munzel-Pedrazzoli, 1970) was used to determine the minimal number of micrographs required to provide stable mean values for statistical comparisons. The sample used in this investigation provided stable means within the 5% (±2·5%) error range. Statistical analysis of the data was performed using the mean values for each of the 5 animals in each experimental group. Initially, an analysis of variance was performed; if this proved significant, comparisons were made between experimental stages using a two-sample *t* test. *P* values of 5% or less were considered significant.

### RESULTS

### *Qualitative observations*

Hemidesmosomes in normal hamster cheek-pouch epithelium had an electron-dense attachment plaque on the cytoplasmic aspect of the inner leaflet of the basal plasma membrane (Fig. 1). Cytoplasmic tonofilaments ran into the attachment plaque. From the outer membrane leaflet fine anchoring filaments ran perpendicularly across the lamina lucida and terminated in the lamina densa. These filaments were found in other parts of the

FIG. 4.—Epithelial–connective tissue junction. Carcinoma stage. The lamina densa is poorly defined and appears to be breaking up. A cytoplasmic protrusion extends into a poorly organized granular connective tissue which contains empty spaces. Hemidesmosomes are infrequent. × 22,500.

lamina lucida, but were more numerous at the sites of the hemidesmosomes. In the region of the hemidesmosomes a localized thickening was found in the lamina lucida, which was invariably much closer to the plasma membrane than to the lamina densa. This thickening, which has been called the sub-basal dense plaque (Briggaman & Wheeler, 1975), the peripheral density (Stern, 1965) and the Haftplat or H line (Komura & Ofuji, 1972), is visible only on perpendicular or near-perpendicular sections of hemidesmosomes. The lamina densa opposite the hemidesmosomes often appeared to be of increased electron density and thickness.

During carcinogenesis (Figs 2–6) a progressive loss of lamina densa and the extrusion of pseudopodia occurred at the epithelial–connective tissue junction (as in Tarin, 1967; Woods & Smith, 1969; White & Gohari, 1981*b*; Smith, 1980). This was accompanied by an apparent decrease in frequency of hemidesmosomes, which became apparent in the later stages of carcinogenesis. The extensive profiles of membranes surrounding the pseudopodia in dysplasia and carcinoma stages were always devoid of hemidesmosomes, but it was common to observe adjacent areas with a complement of hemidesmosomes comparable to that of the normal tissue. There seemed to be a positive correlation between hemidesmosomal loss and poorly differentiated lesions. Individual hemidesmosomes were structurally similar in normal and in carcinogen-treated epithelia, though localized densities on the inner aspect of the cytoplasmic leaflet of the basal plasma membrane became more frequent during carcinogenesis. These structures were most common in carcinomas; they did not possess inserting tonofilaments or more anchoring filaments in the adjacent lamina lucida, and were not related to lamina densa of increased thickness or density. They were, however, seen on membranes both with and without a lamina densa.

*Quantitative observations*

The data are presented in tabular

Fig. 5.—Epithelial–connective tissue junction. Carcinoma stage. A bulbous cytoplasmic projection (P) extends into the amorphous connective tissue through a break in the lamina densa. Hemidesmosomes are frequent and occasional localized densities (arrow) are present on the basal plasma membrane. The epithelial cytoplasm contains peripheral microfilaments (MF) and several microtubules (MT). ×46,000.

(Table I) and histogram (Fig. 7) forms. Table II presents the results of the statistical analysis.

In normal hamster cheek-pouch epithelium 40% of the basal plasma membrane was occupied by hemidesmosomes. In the pre-malignant lesions the relative surface-area decreased progressively to 35% in epithelial hyperplasia and 28% in epithelial dysplasia. The value for squamous-cell carcinoma was only 13%, about 30% of the value in normal epithelium. All alterations were significant at the 5% level or less, with the exception of the comparison between hyperplasia and dysplasia (Table II).

Table I.—*Relative surface area of hemidesmosomes to basal plasma membrane* ($S_{S_{HD,BM}}$) *in hamster cheek pouch carcinogenesis*

| | Normal | Hyperplasia | Dysplasia | Carcinoma |
|---|---|---|---|---|
| | 0·404 | 0·402 | 0·329 | 0·081 |
| | 0·406 | 0·306 | 0·322 | 0·087 |
| | 0·352 | 0·357 | 0·327 | 0·234 |
| | 0·435 | 0·306 | 0·177 | 0·077 |
| | 0·408 | 0·363 | 0·261 | 0·186 |
| Mean | 0·401 | 0·347 | 0·284 | 0·133 |
| s.d. | 0·030 | 0·041 | 0·066 | 0·072 |

Fig. 6.—Epithelial–connective tissue junction. Carcinoma stage. The carcinoma cell lies directly against structureless connective tissue. Basal lamina and hemidesmosomes are totally absent and the cytoplasm contains peripheral fine filaments (arrow). Two dense bodies are present (DB). ×35,000.

Table II.—*Results of statistical analysis of $S_{S_{HD,BM}}$ data (Table I) in hamster cheek pouch carcinogenesis*

| | |
|---|---|
| N *vs* H | $P<0{\cdot}05$ |
| N *vs* D | $P<0{\cdot}01$ |
| N *vs* C | $P<0{\cdot}001$ |
| H *vs* D | N.S. |
| H *vs* C | $P<0{\cdot}001$ |
| D *vs* C | $P<0{\cdot}01$ |

N = normal, H = hyperplasia, D = dysplasia, C = carcinoma.

### DISCUSSION

The ultrastructural characteristics of hemidesmosomes in normal hamster cheek-pouch epithelium are similar to those reported in other epithelia (Farquhar & Palade, 1963, 1965; Susi *et al.*, 1967; Frithiof, 1969; Flickinger, 1970; Geisenheimer & Han, 1971). While there are many subjective descriptions of hemidesmosomes, there are few which have attempted any quantification. Geisenheimer & Han (1971) have estimated the ratio of hemidesmosomes to basal plasma membrane in epithelium from the human gingival crevice, and obtained a value of 27% by planimetry. McNutt (1976) has estimated the mean percentage basal area

FIG. 7.—Histogram demonstrating $Ss_{\mathrm{HD,BM}}$ values in normal and carcinogen-treated epithelium.

occupied by hemidesmosomes, a parameter which is directly comparable with our own stereological results, as 45% in the human epidermis. McNutt also quantified relative hemidesmosomal surface area in seborrhoeic and actinic keratoses, which are benign lesions, and in basal-cell carcinoma, a locally invasive lesion. He obtained values of 42%, 53% and 7% respectively, and statistical comparisons between basal-cell carcinoma and the benign keratoses and control epidermis were significant at the 10% level. Both McNutt's results and our own support the thesis that there is a significant loss of relative hemidesmosomal surface area in malignant lesions, whether they have limited or unlimited invasiveness.

The most important function of hemidesmosomes appears to be to maintain the attachment of the epithelium to the underlying tissues (Briggaman & Wheeler, 1975). The results of the present investigation and those of McNutt imply that the attachment of epithelium to the connective tissue is impaired during experimental carcinogenesis and in basal-cell carcinomas. The area of hemidesmosomal attachment seems to vary from 27 to 45% of the basal plasma membrane in normal epithelia, according to the above-mentioned studies. It would seem reasonable that in normal epithelia a physiological mechanism must exist for the turnover of hemidesmosomes during basal-cell movement on the basal lamina. Such movements must occur after mitosis for a daughter cell to migrate towards the epithelial surface. In the present study no mechanism was detected which might shed light on this problem, other than the increasing presence during carcinogenesis of localized densities on the basal plasma membrane. The lack of inserting tonofilaments and a full condensation of the attachment plaque suggest that these densities might be hemidesmosomes which are undergoing disintegration or removal, or alternatively hemidesmosomes which are forming. It is also possible that they could represent sections through the edges of normal hemidesmosomes. The only accounts of hemidesmosome formation are in migrating epithelial cells during wound healing (Croft & Tarin, 1970; Krawczyk, 1971; Krawczyk & Wilgram, 1973) and these suggest that extracellular events precede intracellular ones, and that the attachment plaque forms before tonofilament insertion. The densities observed in the present study correspond to published micrographs describing the formation of hemidesmosomes.

We have obtained no morphological evidence to elucidate the mechanism for loss which we have described, but reports have been published which suggest such a mechanism. Fukuyama *et al.* (1974) and Scaletta & McCallum (1974) have reported that when stratified squamous epithelium and its adjacent lamina propria are trypsinized dissolution of the basal lamina occurs first, leaving hemidesmosomes as residual densities on the membrane which are subsequently interiorized by endocytosis; this leads to the formation of angular vacuoles with hemidesmosomes initially clearly visible on one side of them. It would appear that the formation of these vacuoles is not due entirely to the action of extrinsic enzymes on the epithelium, since Gona (1970) has reported similar features in tadpole tail fin during resorption *in vivo*, a process which requires lysosomal enzyme activity. Vacuoles incorporating hemidesmosomes, angular or otherwise, were not a feature observed in the present work. The *in vitro* models described above are very drastic, and involve the dissociation of extensive areas of basal plasma membrane. If a similar process occurs *in vivo*, it will obviously be less severe and may involve only focal areas of membrane, producing far fewer vacuoles, which will be seldom encountered ultrastructurally.

Increased levels of enzymes have been described in a variety of neoplasms (Sylven, 1968; Hashimoto *et al.*, 1972, 1973; Strauch, 1972; Yamanishi *et al.*, 1972, 1973; Poole, 1973; Allison, 1974) and increased acid phosphatase reaction product has been demonstrated histochemically (Smith, 1972) and by quantitative cytochemical methods (Gohari, 1977) in hamster cheek-pouch carcinomas. It is possible that an enzymatic mechanism is operative in carcinogenesis *in vivo*, which destroys the lamina densa and adjacent connective tissue (White & Gohari, 1981*b*) as well as the hemidesmosomes; the most likely source seems to be epithelial lysosomal enzymes. However, while Gohari (1977) demonstrated an increased lysosomal component within carcinoma cells, he did not find significant amounts of acid-phosphatase reaction product at the epithelial–connective tissue junction. The most likely mechanism for the destruction at this junction still seems to be enzymatic, but the precise nature of this important alteration awaits elucidation. Pseudopodia have been described previously in premalignant and malignant lesions (Frei, 1962; Tarin, 1967; Frithiof, 1969; Woods & Smith, 1969, 1970; Gould *et al.*, 1975; White & Gohari, 1981*b*; Smith, 1981) and it has been suggested that they may have some role in the release of enzymes which may be responsible for the destruction of the underlying connective tissue. We have described the presence of small electron-dense vacuoles which resemble lysosomal dense bodies (White & Gohari, 1981*b*; White *et al.*, 1981) and these structures may provide the mechanism for these lytic processes. In previous reports (White *et al.*, 1981; White & Gohari, 1981*b*) we have already described the presence of microfilaments in peripheral regions of transforming basal cells. These have also been found in carcinomas by Malech & Lentz (1974), Toh & Muller (1975), Gabbiani *et al.* (1976), McNutt (1976) and Gonda *et al.* (1976). The combination of features of peripheral microfilaments and hemidesmosomal loss has been described in basal keratinocytes during epidermal wound healing (Martinez, 1972), in which cells are actively migrating over collagenous tissue and under blood clot to re-establish epithelial continuity. Thus these features in malignant development strongly suggest an increasing motility of basal cells during this process. Although the details are far from clear, loss of basal lamina and the destruction of adjacent lamina propria during carcinogenesis (White & Gohari, 1981*b*) would seem to provide a less restrictive environment through which malignant epithelial cells could pass. In many areas of the carcinomas there were intact stretches of basal lamina which contained a reduced frequency of hemidesmosomes; below the basal lamina, however, the connective tissue often appeared grossly disorganized. This might signify that connective-tissue loss is brought about by enzymes derived from the inflammatory component, which becomes increasingly significant as carcinogenesis continues. However, if we accept the possibility that inflammatory-cell enzymes alter the hemidesmosomes, it is difficult to see how these can sometimes be affected when the basal lamina is not. A similar argument would apply if the enzymes were of epithelial-cell origin.

The specificity of the changes which we have described is as yet undetermined. Hemidesmosomal loss may be a truly specific feature of malignant development or may simply exist as a result of the inflammatory response. It has already been mentioned that hemidesmosomal loss is a temporary feature of epidermal wound healing, which is also accompanied by inflammation. The acquisition of similar quantitative data for a variety of benign neoplasms and inflammatory conditions is required before any categorical statements can be made regarding the specificity of the changes which we have described.

In conclusion, chemical carcinogenesis in hamster cheek-pouch epithelium is accompanied by a progressive decrease in the relative surface area of hemidesmosomes. Since the principal function of hemidesmosomes is to attach epithelium to connective tissue, this loss may reflect an impairment of the adhesive mechanisms operating in this region. Other ultrastructural features which are present simultaneously, such as peripheral microfilaments, formation of pseudopodia, basal lamina loss and destruction of adjacent connective tissue, may together all act to enable malignant cells to become more motile and invade adjacent tissues.

Part of this work was supported by grants from the Wellcome Trust and the Medical Research Council (F.H.W.) and the Yorkshire branch of the Cancer Research Campaign (K.G.). We are grateful to Professor C. J. Smith for his valuable assistance and encouragement over the years in which this work took place. We would also like to express our thanks to Dr T. M. Mayhew, Department of Human Biology and Anatomy, University of Sheffield, for his help with stereological problems, and to Dr R. A. Dixon, Department of Community Medicine, University of Sheffield, for providing the facilities for the statistical analyses. Maurice Rudland, Neil Cameron, David Thompson, Ray Codd and Maureen Hollingworth provided excellent technical assistance.

### REFERENCES

ALLISON, A. C. (1974) Lysosomes in cancer cells. *J. Clin. Pathol.*, **27** (Suppl. 7), 43.

BRIGGAMAN, R. A. & WHEELER, C. E. (1975) The epidermal-dermal junction. *J. Invest. Dermatol.*, **65**, 71.

CROFT, C. & TARIN, D. (1970) Ultrastructural studies of wound healing in mouse skin. 1. Epithelial behaviour. *J. Anat.*, **106**, 63.

FARQUHAR, M. G. & PALADE, G. E. (1963) Junctional complexes in various epithelia. *J. Cell Biol.*, **17**, 375.

FARQUHAR, M. G. & PALADE, G. E. (1965) Cell junctions in amphibian skin. *J. Cell Biol.*, **26**, 263.

FLICKINGER, C. J. (1970) Extracellular specialisations associated with hemidesmosomes in the fetal rat urogenital sinus. *Anat. Rec.*, **168**, 195.

FREI, J. V. (1962) The fine structure of the basement membrane in epidermal tumours. *J. Cell Biol.*, **15**, 335.

FRITHIOF, L. (1969) Ultrastructure of the basement membrane in normal and hyperplastic human oral epithelium compared with that in preinvasive and invasive carcinoma. *Acta Pathol. Microbiol. Scand.* (Suppl.), 200.

FUKUYAMA, K., BLACK, M. M. & EPSTEIN, W. L. (1974) Ultrastructural studies of newborn rat epidermis after trypsinization. *J. Ultrastruct. Res.*, **46**, 219.

GABBIANI, G., CSANK-BRASSERT, J., SCHNEEBERGER, J.-C., KAPANCI, Y., TRENCHEV, P. & HOLBOROW, E. J. (1976) Contractile proteins in human cancer cells. Immunofluorescent and electron microscopic study. *Am. J. Pathol.*, **83**, 457.

GEISENHEIMER, J. & HAN, S. S. (1971) A quantitative electron microscopic study of desmosomes and hemidesmosomes in human crevicular epithelium. *J. Peridontol.*, **42**, 396.

GOHARI, K. (1977) A quantitative morphological and cytochemical study of the synthetic and metabolic apparatus in hamster cheek pouch epithelium during chemical carcinogenesis. *Ph.D. Thesis, University of Sheffield.*

GONA, A. G. (1970) Changes at the dermo–epidermal junction in resorbing tadpole tail fin *in vivo*. *J. Ultrastruct. Res.*, **30**, 103.

GONDA, M. A., AARONSON, S. A., ELLMORE, N., ZEVE, V. H. & NAGASHIMA, K. (1976) Ultrastructural studies of surface features of human normal and tumour cells in tissue culture by scanning and transmission electron microscopy. *J. Natl Cancer Inst.*, **56**, 245.

GOULD, V. E., MILLER, J. & JAO, W. (1975) Ultrastructure of medullary, intraductal tubular and adenocystic breast carcinomas. Comparative patterns of myoepithelial differentiation and basal lamina deposition. *Am. J. Pathol.*, **78**, 401.

HASHIMOTO, K., YAMANISHI, Y. & DABBOUS, M. K. (1972) Electron microscopic observations of collagenolytic activity of basal cell epithelioma of the skin *in vivo* and *in vitro*. *Cancer Res.*, **32**, 2561.

HASHIMOTO, K., YAMANISHI, Y., MAEYENS, E., DABBOUS, M. K. & KANSAKI, T. (1973) Collagenolytic activities of squamous cell carcinoma of the skin. *Cancer Res.*, **33**, 2790.

KOMURA, J. & OFUJI, S. (1972) Ultrastructure of half desmosomes fixed only in glutaraldehyde. *Dermatologica*, **144**, 35.

KRAWCZYK, W. S. (1971) A pattern of epidermal cell migration during wound healing. *J. Cell Biol.*, **49**, 247.

KRAWCZYK, W. S. & WILGRAM, G. F. (1973) Hemidesmosome and desmosome morphogenesis during epidermal wound healing. *J. Ultrastruct. Res.*, **45**, 93.

MALECH, H. L. & LENTZ, T. L. (1974) Microfilaments in epidermal cancer cells. *J. Cell Biol.*, **60**, 473.

MARTINEZ, I. R. (1972) Fine structural studies of migrating epithelial cells following incision wounds. In *Epidermal Wound Healing*. Ed.

Maibach & Rovee. Chicago: Year Book Medical Publishers Inc. p. 323.

MAYHEW, T. M. (1979) Basic stereological relationships for quantitative microscopical anatomy—a simple systematic approach. *J. Anat.*, **129,** 95.

MCNUTT, N. S. (1976) Ultrastructural comparison of the interface between epithelium and stroma in basal cell carcinoma and control human skin. *Lab. Invest.*, **35,** 132.

PINDBORG, J. J., RENSTRUP, G., POULSON, H. E. & SILVERMAN, S., JR (1963) Studies in oral leukoplakias. V. Clinical and histologic signs of malignancy. *Acta Odont. Scand.*, **21,** 407.

POOLE, A. R. (1973) Tumour lysosomal enzymes and invasive growth. In *Lysosomes in Biology and Pathology, Vol. 3.* Ed. Dingle. Amsterdam: North Holland. p. 303.

SCALETTA, L. J. & MACCALLUM, D. K. (1974) A fine structural study of human oral epithelium separated from the lamina propria by enzymatic action. *Am. J. Anat.*, **140,** 383.

SCHROEDER, H. E. & MUNZEL-PEDRAZZOLI, S. (1970) Application of sterologic methods to stratified gingival epithelia. *J. Microsc.* (*Oxf.*), **92,** 179.

SMITH, C. J. (1972) Variation in histochemical properties of hydrolytic enzymes in oral pre-cancerous conditions. *J. Dent. Res.*, **51,** 308.

SMITH, C. J. & PINDBORG, J. J. (1969) *Histological Grading of Oral Epithelia Atypia by the use of Photographic Standards.* Copenhagen: C. Hamburgers' Bogtrykkeri.

SMITH, C. J. (1981) Connective tissue influences on epithelial malignancy and premalignancy. In *Oral Premalignancy. Proc. 1st Dows Symposium.* Ed. Mackenzie *et al.* Iowa City: University of Iowa Press. p. 148.

STAEHELIN, L. A. (1974) Structure and function of intercellular junctions. *Int. Rev. Cytol.*, **39,** 191.

STERN, I. B. (1965) Electron microscopic observations of oral epithelium. I. Basal cells and the basement membrane. *Periodontics*, **3,** 224.

STRAUCH, L. (1972) The role of collagenases in tumour invasion. In *Tissue Interactions in Carcinogenesis.* Ed. Tarin. London: Academic Press. p. 399.

STRÄULI, P. & WEISS, L. (1977) Cell locomotion and tumour penetration. *Eur. J. Cancer*, **13,** 1.

SUSI, F. R., BELT, W. D. & KELLY, J. W. (1967) Fine structure of fibrillar complexes associated with the basement membrane in human oral mucosa. *J. Cell Biol.*, **34,** 686.

SYLVEN, B. (1968) Lysosomal enzyme activity in the interstitial fluid of solid mouse tumour transplants. *Eur. J. Cancer*, **4,** 463.

TARIN, D. (1967) Sequential electron microscopical study of experimental mouse skin carcinogenesis. *Int. J. Cancer*, **2,** 195.

TOH, B. H. & MULLER, H. K. (1975) Smooth muscle-associated antigen in experimental cutaneous squamous cell carcinoma, keratoacanthoma and papilloma. *Cancer Res.*, **35,** 3741.

WHITE, F. H. (1978) A stereological study of hamster cheek pouch epithelium: Ultrastructural changes in differentiation and chemical carcinogenesis. *Ph.D. Thesis, University of Sheffield.*

WHITE, F. H. & GOHARI, K. (1981*a*) The ultrastructural morphology of hamster cheek pouch epithelium. *Archs. Oral Biol.*, **26,** 563.

WHITE, F. H. & GOHARI, K. (1981*b*) A quantitative study of lamina densa alterations in hamster cheek pouch carcinogenesis. *J. Pathol.* (In press).

WHITE, F. H., GOHARI, K. & SMITH, C. J. (1981) Histological and ultrastructural morphology of 7,12 dimethylbenz(α)anthracene carcinogenesis in hamster cheek pouch epithelium. *Diagnostic Histopath.* (In press).

WOODS, D. A. & SMITH, C. J. (1969) Ultrastructure of the dermal–epidermal junction in experimentally-induced tumours and human oral lesions. *J. Invest. Dermatol.*, **52,** 259.

WOODS, D. A. & SMITH, C. J. (1970) Ultrastructure and development of epithelial cell pseudopodia in chemically induced pre-malignant lesions of the hamster cheek pouch. *Exp. Molec. Pathol.*, **12,** 160.

YAMANISHI, Y., DABBOUS, M. K. & HASHIMOTO, K. (1972) Effect of collagenolytic activity in basal cell epithelioma of the skin on reconstituted collagen and physical properties and kinetics of the crude enzyme. *Cancer Res.*, **32,** 2551.

YAMANISHI, Y., MAEYENS, E., DABBOUS, M. K., OHYAMA, H. & HASHIMOTO, K. (1973) Collagenolytic activity in malignant melanoma. Physiochemical studies. *Cancer Res.*, **22,** 2507.